# The interrelation between major depressive symptoms and ambivalence over emotional expression among college students: a network perspective on gender differences

**DOI:** 10.3389/fpsyt.2025.1658159

**Published:** 2025-09-17

**Authors:** Xiangxiang Chen

**Affiliations:** The Affiliated Brain Hospital of Nanjing Medical University, Nanjing, China

**Keywords:** depression, ambivalence over emotional expression, college students, gender difference, network analysis

## Abstract

**Introduction:**

Major depressive disorder is a common and severe mental disorder among college students. Meanwhile, depressive symptoms and ambivalence over emotional expression are closely related, while little research explores their bidirectional relationship. To address this gap, the current study employed a network approach to identify the interrelation between depressive symptoms and ambivalence over emotional expression among college students.

**Methods:**

Initially, 2,103 college students were recruited and completed the patient health questionnaire (PHQ) -9 and the ambivalence over emotional expression questionnaire (AEQ). In the final analysis, 1,362 college students passed the attention check and were included (674 females; age: *Mean* = 18.61, *SD* = 0.84). The symptom network approach was employed to explore the interrelation between depressive symptoms and ambivalence over emotional expression, as well as to explore the gender difference between symptom networks.

**Results:**

The strongest edges between depression and ambivalence over emotional expression were observed between “concentration difficulties” (PHQ7) and “emotional rumination” (AEQ1), as well as between “guilt” (PHQ6) and “regret expressing” (AEQ5) in the overall sample. The edge between “inhibit positive emotion expression” (AEQ3) and “inhibit negative emotion expression” (AEQ4) was the strongest edge weight in male and female networks. For bridging symptoms, “concentration difficulties” (PHQ7), “emotional rumination” (AEQ1), “guilt” (PHQ6), and “regret expressing” (AEQ5) were the biggest bridging symptoms (*Z* score above 1) that linked depression symptoms and ambivalence over emotional expression. Between gender networks, “guilt” (PHQ6) was the common and strongest bridging symptom (*Z* score above 1) in both male and female networks. Network robustness and stability were also estimated.

**Conclusion:**

The current study provides a new perspective on the interrelation between depressive symptoms and ambivalence over emotional expression, as well as examines the gender difference. In light of the findings, further intervention, such as cognitive control training or mindfulness-based interventions that focus on bridging symptoms, may disassociate the interrelation between depression and ambivalence over emotional expression.

## 1 Introduction

Major depressive disorder is a common mental illness characterized by many symptoms, including low mood, loss of interest and appetite, and a sense of worthlessness (1, 2). College students have been considered a high-risk group for developing depression, as the lifetime risk of depression is estimated to be around 15-18% worldwide, with prevalence peaking in the twenties and thirties (3, 4). The latest report on National Mental Health Development in China (2023-2024), released in 2025, involving 60,782 young adults aged 16-28, pointed out that depression levels peak at 18-24 (similar age with college studnets), and that women are at a higher risk of depression (5). A meta-analysis involving 32 cross-sectional studies showed that the summary prevalence of Chinese college students suffering from depressive symptoms was 34.7% (6). Depression is not only associated with a series of mental and physical health problems, including anxiety, sleep disturbance, and suicide, but also has a lasting impact on their psychological state in late adulthood (7–10). Given the prevalence and serious consequences of depression in college students, identifying risk factors related to depression may help develop relevant interventions.

A rich body of studies have identify that ambivalence over emotional expression (AEE) is closely related to depressive symptoms, suggesting that when helping individuals to cope with their depressive symptoms, attention should also be paid to their expressive styles as it is the ambivalence about one’s expression that results in mental distress (e.g., 7, 11, 12). However, a large gap still exists in current knowledge of the links and pathways between depression and AEE. This current study therefore aims to clarify the association mechanism between depression and AEE in a group of Chinese college students of different genders.

Ambivalence over emotional expression is defined as the conscious inhibition of emotional expression despite a genuine desire to communicate affect (13, 14). This phenomenon encompasses the desire to express but fear of such self-expression, the expression that is incongruent with one’s true affective state, or the experience of regret, shame, or even self-criticism following emotional expression. Based on the perspective of cognitive appraisal theory (15), when an individual evaluates the expression of emotion as likely to yield negative consequences (e.g., rejection or punishment), they may actively suppress emotional expression. In line with this, a study conducted by Krause et al. (16) and Cabecinha-Alati et al. (17) have demonstrated that individuals with a history of abuse and parental punishment during childhood are more prone to chronic emotional inhibition in adulthood, as such experiences lead them to develop a rigid cognitive schema that any emotional expression will inevitably elicit punitive responses. King and Emmons (13) and Rothman et al. (18) posit that AEE may serve as a short-term protective mechanism against social rejection. As a manifestation of emotional dysregulation, however, AEE has been shown to be linked with a variety of personal consequences in the long term, such as difficulty accepting support from others, lower level of well-being, and higher levels of experiencing depressive symptoms (19–21).

As for the link between AEE and depression, on the one side, empirical evidence has corroborated AEE’s pathogenic role in depression. Specifically, a gender-differentiated analysis designed by Kunst et al. (21) revealed that women heightened their ambivalence mediated depression risk to express sadness. Lee (22) further postulates that individuals who are ambivalent about expressing emotions create significant barriers for others to perceive their distress signals, thereby constraining access to professional help and subsequently aggravating the progression and severity of depressive symptomatology. In another study involving Singaporean participants, it has been demonstrated that AEE may exacerbate depressive symptoms by amplifying somatization tendencies (23). On the other side, depression can inversely reinforce one’s avoidance of expressing emotions freely, as the robust association between these two mental health problems is also evidenced by significantly higher AEE levels reported among individuals with major depressive disorder compared to non-depressed controls (11). This phenomenon may stem from depressed individuals’ heightened sensitivity to perceived criticism and rejection, coupled with their fear of being overwhelmed by distressing emotions should they openly express their psychological pain (24). Thus, individuals with depression tend to demonstrate emotional avoidance even when needing interpersonal interaction. Crucially, this maladaptive pattern endures among clinically recovered individuals who continue to exhibit a preference for suppressing emotional expression (25).

Taken together, previous empirical research collectively demonstrates a bidirectional reinforcement relationship between AEE and depression. Specifically, individuals with AEE become trapped in emotional regulation difficulties, unwilling to express their emotions, thereby exacerbating mental disorders (26). Conversely, depressed individuals tend to avoid sharing their emotional pain with others, which in turn intensifies emotional expression conflicts. However, current understanding in this field remains unclear about precisely how these two mental health problems intertwine. This gap may stem from traditional research approaches primarily using total scores to examine latent variable relationships. In contrast, network analysis offers a novel perspective (27), showing that each disorder comprises an interconnected system of symptoms, and that relationships between disorders can be clearly revealed via symptom-to-symptom connections and bridge symptoms (i.e., one symptom within a specific disorder that is closely linked to another disorder’s symptoms). For instance, through symptom network analysis of depression, Tao et al. (28) and Liang et al. (29) both discovered that “sadness mood” plays the most central role in adolescents’ depressive symptom network structure, which implies that interventions specifically targeting this core symptom may yield optimal therapeutic outcomes for depression recovery. Additionally, a study investigating maladaptive emotion regulation strategies among college students found that “controlling emotions by not expressing them” from expressive suppression had significant positive correlations with “symptom rumination” (refers to the repeated thinking about the causes and may result of depressed mood) from rumination (30). When gender factors were considered, the study conducted by Kunst et al. (21) suggested that women’s typically higher depression levels may be mediated by their ambivalent expression of sadness and anger. However, it should be noted that only approximately half of the study participants were college students.

In summary, although there is increasing evidence that AEE is associated with depressive symptoms, few studies have been conducted from the perspective of network analysis to understand how depression is associated with AEE and clarify the role of gender. To fill this gap and help to develop effective interventions for depression, this study focused on (1) exploring the network structure between depressive symptoms and AEE, and (2) clarifying the gender differences in the network structure.

## 2 Method

### 2.1 Procedures and participants

From October to December 2023, a total of 2,103 college students from *Nanjing* participated in the survey via Wenjuanxing, a widely used online platform in China (https://www.wjx.cn). All participants completed the Patient Health Questionnaire-9 and Ambivalence over Emotional Expression Questionnaire. While 229 participants were excluded for not passing the attention check (item: “*Please select the tiger.*”), and 512 participants were excluded for failing to stay at one item for less than 2 seconds (31). Finally, a total of 1,362 participants (valid response rate:64.8%; 688 men and 674 women; *Mean _age_* =18.61, *SD _age_* =0.84) were included in network analysis.

Before the survey, all participants gave electronic informed consent and were informed of their right to withdraw at any time. The Ethics Committee of Nanjing Brain Hospital reviewed and approved the present study (Reference number: 2025-KY081-01).

### 2.2 Measures

#### 2.2.1 Patient health questionnaire-9

The PHQ-9 was employed to assess depressive symptoms (32). Participants evaluated how often they experienced depressive symptoms (e.g., “depressed mood”, “fatigue”, and “guilt”) over the past two weeks using a four-point Likert scale (0=*Not at all*, 3=*almost everyday*). Higher total scores reflect greater severity of depression. In this study, the Cronbach’s *α* was 0.90.

#### 2.2.2 Ambivalence over emotional expression questionnaire

The AEQ was used to assess individuals’ hesitation and internal conflict regarding emotional expression. Participants rated their agreement with each statement on a 7-point Likert scale. The Chinese version of AEQ consists of 23 items (33), which includs five dimensions: emotional rumination (e.g., “I want to express my emotions honestly, but I am afraid that it may cause me embarrassment or hurt”; Cronbach’s *α* =0.90), inhibit negative (e.g., “I think about acting when I am angry but I try not to”; Cronbach’s *α* =0.90), inhibit positive (e.g., “Often I find that I am not able to tell others how much they really mean to me”; Cronbach’s *α*=0.93) emotions expression, desire to be understood (e.g., “I try to suppress my anger, but I would like other people to know how I feel”; Cronbach’s *α* =0.94), and regret expressing (e.g., “I often cannot bring myself to express what I am really feeling”; Cronbach’s *α* =0.93). Higher scores reflect greater emotional ambivalence. In this study, the Cronbach’s *α* of the full scale was 0.93.

### 2.3 Statistical analysis

All analyses were performed using R software (version 4.3.2; 34). Descriptive statistics were conducted using the *describe* function in the *psych* package (v2.3.9) for demographic characteristics (e.g., age, only-child status, and parental marital status). Mean scores for study variables (e.g., depression and AEQ scores) were subsequently calculated for the overall sample, and the gender difference in depression and AEQ was examined using independent-samples t-tests with Cohen’s *d* as the effect size between men and women.

Before estimating the network, we examined univariate distributions (skewness, kurtosis) for all variables. Because several PHQ items showed mild–moderate positive skew, we reran the whole network using Spearman instead of Pearson correlations as a sensitivity check. The Spearman- and Pearson-based correlation matrices were nearly identical (*r*=0.9796, *p*=.001), indicating that the network structure is robust to slight departures from normality and that using Pearson correlations is appropriate. Detailed procedures are provided below:

#### 2.3.1 Network structure and centrality estimation

In this study, Depression-AEQ were estimated networks for the overall, men, and women samples, respectively. Network estimation and visualization were conducted using the R packages *bootnet* 1.4.3 and *qgraph* 1.6.9 (35, 36). The network was constructed with the *estimateNetwork* function in the R package *bootnet* 1.4.3, using the Extended Bayesian Information Criterion (EBIC) graphical least absolute shrinkage and selection operator (LASSO) method. This method estimates the partial correlations between variables and shrinks weak edges toward zero to produce a sparse and interpretable network (35). In the network visualized, nodes represented the symptoms of depression and dimensions of AEQ, and edges between nodes represented the relationships. Blue (red) edges indicated positive (negative) relationships, with thicker edges denoting stronger relationships (37).

Expected Influence (EI) and bridge EI centrality for each node were computed using the *centralityPlot* function in the R package *qgraph* 1.6.9 (38). EI represents the sum of all edge weights connected to a given node among the disorder community, reflecting its overall impact within the network. On the other hand, bridge EI quantifies the total strength of connections between a node within one disorder community and other nodes in another disorder community, highlighting its role in linking distinct disorder symptom clusters (39). In line with prior literature, nodes with standardized centrality scores above 1 were considered as central symptoms (28).

#### 2.3.2 Network stability and accuracy

The accuracy and stability of all three networks were evaluated using the R package *bootnet* 1.4.3 (35). The nonparametric bootstrapping test was applied to estimate 95% confidence intervals (CIs) for edge weights; narrow CIs indicated reliable edge ranking. Centrality stability was assessed through a case-dropping bootstrap procedure using the *corStability* function, which yielded the correlation stability coefficient (CS-C). This coefficient reflects the maximum proportion of the sample that can be removed while still maintaining, with 95% probability, a correlation of at least 0.7 between the centrality estimates from the full and reduced samples. The CS-C values above 0.25, 0.50, and 0.75 indicate acceptable, good, and excellent stability, respectively (40). Additionally, bootstrapped difference tests were conducted using the *differenceTest* function to assess the statistical significance of differences between edge weights and node centrality indices.

#### 2.3.3 Network comparison test

Gender differences were explored using the R package *NetworkComparisonTest* (NCT; 41). NCT offers four primary testing approaches: the network structure invariance test evaluates the differences in network structures; the global strength invariance test compares the differences in the total edge strengths across groups; the edge strength invariance test detects variations in the local edge strengths; and the centrality invariance test measures group differences in node centralities.

## 3 Results

### 3.1 Descriptive statistics and group differences

**Table 1** displays the descriptive statistics and gender comparisons for demographic and main variables, with gender differences tested using independent-samples t-tests for continuous variables and chi-square tests for categorical variables. Among the participants, 24.3% were only children, with significantly more men (32.3%) than women (16.2%) reporting this status (*p* <.001). Most participants reported that their parents were not divorced (86.2%), and no gender difference was observed for parental marital status (*p* =.125).

**Table 1:** Descriptive Statistics and Gender Differences.

Variable	Total sample	Gender		*P*		Cohen’s*d*
		Men (*N*=688)	Women (*N*=674)			
Demographic variables						
Age (in years old)	18.61 (0.84)	18.63 (0.84)	18.59 (0.83)	.447		-0.04
Only child						
Yes	331 (24.3%)	222 (32.3%)	109 (16.2%)	<.001	***	——
No	1031 (75.7%)	466 (67.7%)	565 (83.8%)			
Parental marriage						
Not divorced	1174 (86.2%)	591 (85.9%)	583 (86.5%)	.125		——
Not divorced but living apart long-term	24 (1.8%)	12 (1.7%)	12 (1.8%)			
Not divorced but one or both parents are deceased	32 (2.3%)	22 (3.2%)	9 (1.3%)			
Divorced	133 (9.8%)	63 (9.2%)	70 (10.4%)			
Study variables						
Depression (PHQ)	4.67 (4.68)	4.47 (4.81)	4.87 (4.54)	.113		0.09
PHQ1: Anhedonia	1.63 (0.69)	1.60 (0.70)	1.66 (0.67)	.126		0.08
PHQ2: Depressed or sad mood	1.48 (0.63)	1.47 (0.65)	1.48 (0.62)	.742		0.02
PHQ3: Sleep difficulties	1.66 (0.83)	1.61 (0.81)	1.72 (0.85)	.013	*	0.14
PHQ4: Fatigue	1.81 (0.82)	1.75 (0.81)	1.88 (0.82)	.006	**	0.15
PHQ5: Appetite changes	1.58 (0.73)	1.51 (0.72)	1.64 (0.74)	.001	**	0.18
PHQ6: Guilt	1.45 (0.67)	1.46 (0.71)	1.43 (0.63)	.424		–0.04
PHQ7: Concentration difficulties	1.58 (0.75)	1.58 (0.76)	1.58 (0.75)	.940		0.00
PHQ8: Motor disturbances	1.34 (0.62)	1.34 (0.63)	1.34 (0.61)	.975		0.00
PHQ9: Suicide ideation	1.15 (0.43)	1.15 (0.44)	1.15 (0.41)	.802		–0.01
Ambivalence over Emotional Expression (AEQ)	3.80 (1.51)	3.75 (1.57)	3.85 (1.44)	.198		0.07
AEQ1: Emotional rumination	3.57 (1.66)	3.46 (1.71)	3.68 (1.60)	.015	*	0.13
AEQ2: Desire to be understood	3.88 (1.62)	3.81 (1.70)	3.96 (1.53)	.090		0.09
AEQ3: Inhibit positive emotion expression	3.82 (1.62)	3.81 (1.68)	3.82 (1.56)	.869		0.01
AEQ4: Inhibit negative emotion expression	3.95 (1.59)	3.94 (1.65)	3.96 (1.53)	.820		0.01
AEQ5: Regret expressing	3.74 (1.66)	3.68 (1.72)	3.80 (1.61)	.161		0.08

The chi-square test or independent samples t-test was used to compare the groups.

*, p < .05; **, p < .01; ***, p < .001.

In terms of main variables, women participants reported higher average scores than men on depression and AEQ symptoms, although the difference was not statistically significant *(p* ≥.113*)*. However, significant gender differences were observed in several specific depressive symptoms. Women reported greater *“sleep difficulties”* (PHQ3; *p*=.013), *“fatigue”* (PHQ4; *p* =.006), and *“appetite changes”* (PHQ5; *p*=.001), all with small effect sizes (Cohen’s *d*=0.14–0.18). Moreover, women reported significantly greater difficulty in experiencing *“emotional rumination”* (AEQ1; *p*=.015), while no other AEQ symptoms showed significant gender differences.

### 3.2 Network structures and centrality for the full sample

**Figure 1:**
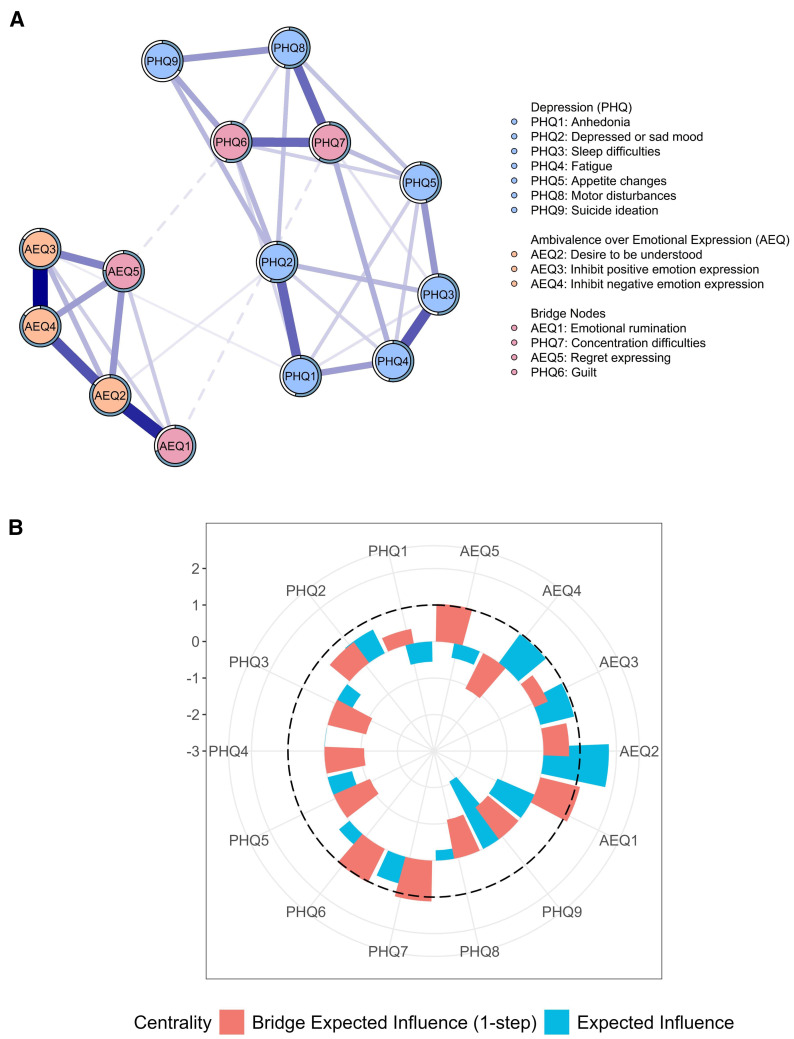
Depression–AEQ network structures **(A)** and node centrality **(B)** of AEQ and depression symptoms. All edges in the network were blue, indicating positive associations; thicker lines represent stronger connections. Edges through bridge nodes were presented in dashed lines.

**Figure 1A** shows the comorbidity network of depression and AEQ (see edge-weight matrices in **Supplementary Table S1**). The depression-AEQ network was depicted as two clusters, with the clusters connected by several bridging edges that represent significant associations between symptoms of depression and dimensions of AEQ.

In the cluster of depression symptoms, the edge between “*sleep difficulties*” (PHQ3) and “*fatigue*” (PHQ4) has the strongest edge weight (*r*=0.326), followed by the edge between “*anhedonia*” (PHQ1) and “*depressed or sad mood*” (PHQ2; *r*=0.304). In the cluster of AEQ dimensions, the strongest edge weight was between “*inhibit positive emotion expression*” (AEQ3) and “*inhibit negative emotion expression*” (AEQ4; *r*=0.500), followed by the edge between “*emotional rumination*” (AEQ1) and “*desire to be understood*” (AEQ2; *r*=0.427).

Regarding centrality (see **Supplementary Table S1**, **Figure 1**), “*desire to be understood*” (AEQ2) had the highest EI centrality and was identified as the most central symptom in the depression-AEQ network. Additionally, the strongest edges between depression and ambivalence over emotional expression were observed between “*concentration difficulties*” (PHQ7) and “*emotional rumination*” (AEQ1), as well as between “*guilt*” (PHQ6) and “*regret expressing*” (AEQ5). Among all symptoms, PHQ7, PHQ6, AEQ1, and AEQ5 exhibited the highest bridge EI values (all=1.030 to 1.119), suggesting their pivotal role in transmitting activation across the depression and AEQ sub-networks.

### 3.3 Network structures and centrality for men and women

**Figure 2:**
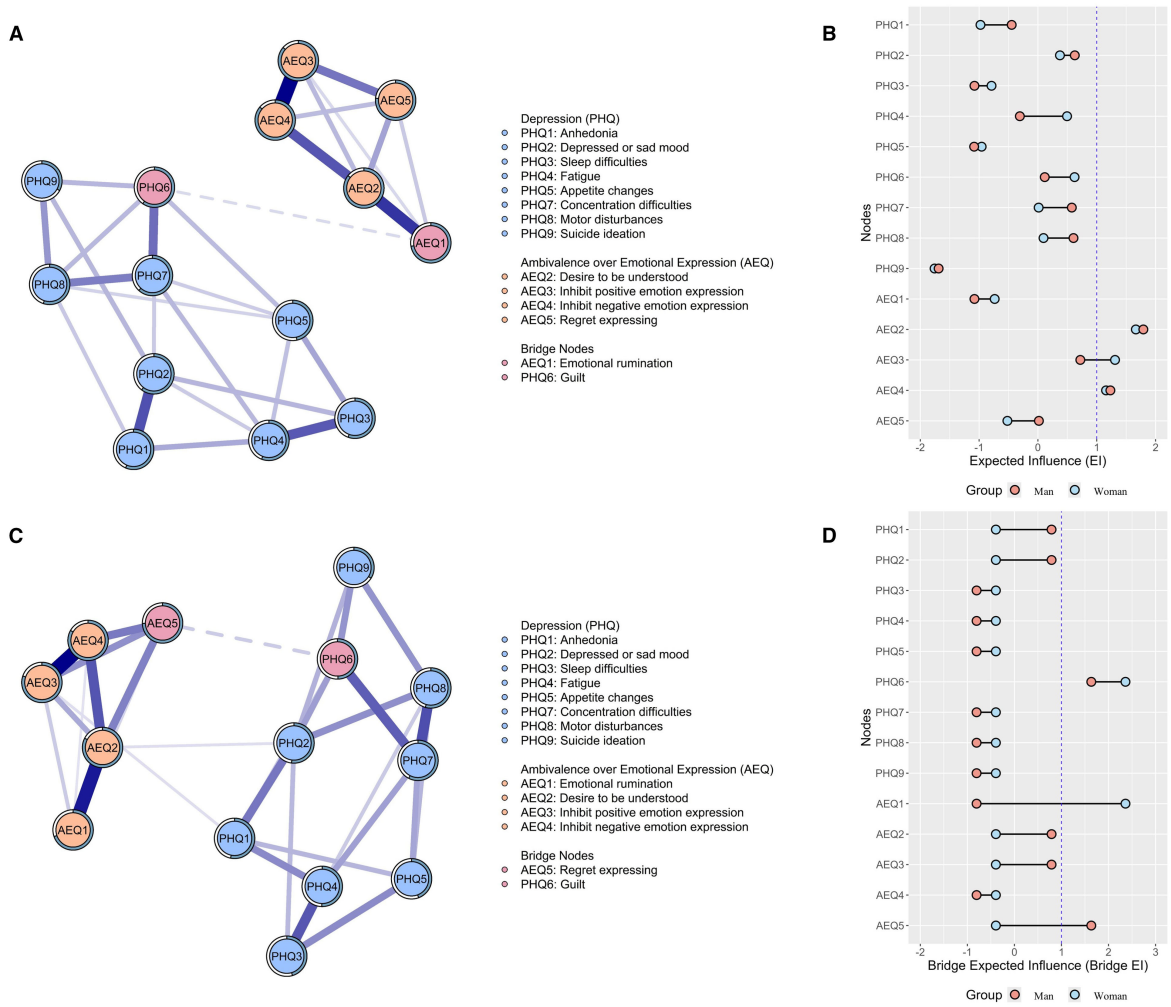
Depression–AEQ network structures and node centrality among men (**(A, B)** and women **(C, D)**. In parts **(A, C)**, all edges in the network were blue, indicating positive associations; thicker lines represent stronger connections. Edges through bridge nodes were presented in dashed lines.

**Figure 2A** and 2C show the separate network of men and women samples (see edge-weight matrices in **Supplementary Tables S2, S3**). In the men network, the edge between “*inhibit positive emotion expression*” (AEQ3) and “*inhibit negative emotion expression*” (AEQ4) has the strongest edge weight (*r*=0.530), followed by the edge between “*emotional rumination*” (AEQ1) and “*desire to be understood*” (AEQ2; *r*=0.422). Similarly, in the women’s network, the strongest edge weight was between AEQ3 and AEQ4 (*r*=0.465), followed by the edge between AEQ1 and AEQ2 (*r*=0.419).

Considering node centrality, “*desire to be understood*” (AEQ2), “*inhibit positive emotion expression*” (AEQ3), and “*inhibit negative emotion expression*” (AEQ4; EI ≥ 1.158) were more central in the men network, whereas the women network showed a particularly high EI only for “*desire to be understood*” (AEQ2; EI=1.792) and “*inhibit negative emotion expression*” (AEQ4; EI=1.232). Moreover, analysis of bridge EI revealed that “*guilt*” (PHQ6) and “*emotional rumination*” (AEQ1) were identified as the main bridging nodes that link depressive symptoms community and ambivalence over emotional expression community in the men’s network, whereas “*guilt*” (PHQ6) and “*regret expressing*” (AEQ5) played a pivotal bridging role in the women’s network.

### 3.4 Group differences in network

The NCT tests indicated no significant difference was found in the network structure (*p*=.28) or the global strength (S=0.159, *p*=.65). However, several edge weights significantly differed between genders (details in **Figure 2** and **Supplementary Table S4**). Specifically, compared to the men group, the women group exhibited stronger edge weights on “*emotional rumination*-*inhibit negative emotion expression*” (AEQ1-AEQ4; diff=0.053, *p* =.010), “*depressed or sad mood*-*motor disturbances*” (PHQ2-PHQ8; diff=0.205, *p* <.059), as well as “*guilt* -*regret expressing*” (PHQ6-AEQ5; diff=0.092, *p* =.030). Conversely, the edge “*anhedonia*-*motor disturbances*” was marginally stronger in men (PHQ1-PHQ8; diff=0.114, *p*=.079). Regarding centrality, there is no significant gender difference in the EI values of nodes (see **Supplementary Table S5**). However, a significant gender difference was observed in the bridge EI of “*regret expressing*” (AEQ5), with women exhibiting higher values than men (diff=0.092, *p*=.040; see **Supplementary Table S5**).

### 3.5 Network accuracy and stability

Bootstrapped analyses of edge weights (**Supplementary Figure S1**) showed that the edge weights in the depression-AEQ networks for the total, men, and women participants demonstrated acceptable accuracy, with narrow 95% *CI*s. Case-dropping bootstrap analyses (**Supplementary Figure S2**) further indicated that the EI centrality values were stable across the total sample (CS-C=0.67), men participants (CS-C=0.52), and women participants (CS-C=0.44) networks. These results suggest the maximum extent to which the sample size can be reduced while preserving the network structure’s EI stability. However, the bridge EI centrality values were unstable in all three networks (CS-Cs below 0.25) and should therefore be interpreted cautiously. In addition, **Supplementary Figures S3**, **S4** display the results of bootstrapped difference tests for edge weights and centrality indices (i.e., EI and bridge EI), respectively. In all three networks, most edge weights and symptoms EIs were significantly different from one another.

## 4 Discussion

The current study employed the network approach to explore the interrelation between depressive symptoms and ambivalence over emotional expression (AEE), as well as to examine the gender difference between the network structures. Some findings are worth discussing.

Firstly, the current results found no significant difference between the overall scores of depression and AEE, while three depressive symptoms and one dimension of AEE revealed notable differences. Consistent with previous studies (42, 43), women reported significantly higher sleep difficulties, fatigue, and appetite changes than their men counterpart. It is noted that these three depressive symptoms are attributed to somatic-affective symptoms, potentially reflecting gender-based physiological or psychosocial vulnerabilities (44). Interestingly, despite these symptom-specific differences, the absence of a significant difference in overall depressive severity suggests that men and women may experience depression through different symptoms rather than differing in overall depressive severity (45). Similarly, while the emotional rumination dimension of AEE was significantly higher in women, no gender difference was found in the overall ambivalence score. This finding suggests that although women tend to engage more in maladaptive emotional processing (46) and are prone to experience rumination (47), their overall emotional ambivalence is comparable to that of men. Taken together, findings highlight the importance of examining gender differences at both the symptom and subscale levels, rather than relying solely on the overall scores.

Regarding bridge centrality symptoms between the network of depression and AEE, “*concentration difficulties*” and “*emotional rumination*” showed the strongest cross-domain edge in the overall sample. This finding highlights the strong interplay between cognitive disruption and maladaptive emotional processing in individuals experiencing depressive symptoms (48, 49). Emotional rumination, marked by persistent focus on negative affect and internal conflict over emotional expression (50), may interfere with attentional control and executive functioning (51), thereby intensifying difficulties with concentration. In addition, longitudinal studies revealed that impaired concentration may limit the capacity to disengage from repetitive negative thoughts (52), further perpetuating emotional rumination (53). The bidirectional relation observed indicates a reinforcing cognitive-affective cycle, wherein rumination and attentional impairments dynamically interact to perpetuate one another, thereby contributing to the persistence of depressive symptomatology. This reciprocal mechanism may constitute a critical pathway through which depressive states and ambivalence toward emotional expression become mutually reinforcing, intensifying the severity and duration of affective disturbances.

Notably, both “*concentration difficulties*” and “*emotional rumination*” exhibited the highest bridge expected influence values, highlighting their central roles in transmitting activation between depression and emotional ambivalence, which suggests that cognitive impairments and maladaptive emotional rumination function as critical bridge symptoms linking the two mental problems. These findings point to the theoretical utility of conceptualizing bridge symptoms as transdiagnostic connectors that maintain symptom comorbidity and emotional dysregulation across diagnostic boundaries (39). Clinically, this implies that targeting these comorbidity mechanisms may have cascading effects across symptom domains, offering more efficient intervention strategies for individuals presenting with comorbid emotional and cognitive dysfunction. Accordingly, interventions that specifically target these mechanisms may be particularly effective in disrupting the feedback cycle between depressive symptoms and emotional ambivalence. For instance, cognitive control training (54) protocols to strengthen executive function could enhance individuals’ ability to disengage from perseverative negative thinking, thereby reducing concentration difficulties and emotional rumination. In the case of rumination, enhanced working memory updating and inhibitory control can reduce the persistence of repetitive negative thinking by replacing maladaptive thought patterns with more adaptive content. Likewise, for concentration difficulties, improved attentional shifting can facilitate the rapid disengagement from emotionally charged distractions, enabling sustained attention on academic or daily tasks. To sum up, the identification of bridge symptoms further supports the value of transdiagnostic approaches (55) that address shared cognitive-affective processes. Interventions such as cognitive control training (54) or mindfulness-based interventions (56), which aim to improve executive functioning and reduce maladaptive emotion regulation, may thus represent a promising avenue for addressing the complex interplay between cognitive dysfunction and emotional ambivalence in depression.

For the network structure difference between genders, the strongest connections observed in both men’s and women’s networks were the edge between “*inhibit positive emotion expression*” and “*inhibit negative emotional expression*”, as well as between “*emotional rumination*” and “*desire to be understood*”. This pattern aligns with the dual-process model of emotion regulation (57), which posits that maladaptive outcomes arise from impairments in both bottom-up emotional reactivity and top-down regulatory control. In particular, impairments in expressive inhibition and deficits in cognitive-affective integration may jointly underlie maladaptive emotional processing, pointing to fundamental mechanisms that appear to function consistently across genders (58) despite possible variations in emotional socialization (59). The convergence of these edges across men’s and women’s networks suggests the existence of a shared latent structure underpinning emotional ambivalence, in which suppression and unmet interpersonal emotional needs are tightly interwoven. In summary, regardless of gender, individuals experiencing depressive symptoms may struggle with both internal restraint in emotional expression and a simultaneous yearning for emotional validation, forming a paradox that sustains psychological distress.

Analysis of the bridge’s expected influence revealed distinct gender-specific patterns within the depression and emotional ambivalence network. Among men, “*guilt*” and “*emotional rumination*” play central bridging nodes, indicating that internalized negative affect and perseverative emotional processing may serve as key mechanisms linking depressive symptoms with emotional ambivalence. This suggests a profile of emotional dysregulation in which guilt becomes a cognitive anchor for ruminative cycles (60), potentially reinforcing depressive symptomatology through unresolved self-blame and suppression. In contrast, the women’s network was primarily characterized by the bridging roles of “*guilt*” and “*regret expressing*,” suggesting that interpersonal emotional conflict, particularly distress associated with past emotional disclosures (61), may play a more prominent role in the interplay between depressive states and ambivalence over emotional expression. These gender-specific patterns may reflect broader socio-developmental trajectories shaped by emotional socialization processes (45, 46), whereby men are typically socialized to adopt stoic or emotionally self-restrained styles that favor internalization, whereas women are often encouraged to express emotions yet may face social sanctions for excessive expression, potentially fostering relational ambivalence. Related to the current study, such socialization processes not only shape emotional coping styles (62) but may also influence the development of specific bridge symptoms within psychopathological networks. These findings may reflect gendered patterns of emotional socialization, wherein men are more likely to cognitively internalize emotional distress, while women may be more sensitive to the relational consequences of emotional expression. To sum up, the current study found that distinctions highlight the need for gender-informed models of emotional functioning in the context of depression.

### 4.1 Implications

Some implications should be noted. Firstly, the identification of “*concentration difficulties*” and “*emotional rumination*” as key bridge symptoms suggests that by focusing treatment efforts on these nodes, clinicians may effectively reduce both depressive symptom severity and ambivalence over emotional expression, potentially disrupting the reinforcing cycle between cognitive and emotional dysregulation. Secondly, these findings highlight the importance of adopting gender-informed models of emotional functioning in the context of depression. Future interventions may benefit from addressing these divergent pathways by tailoring treatment components accordingly. For example, self-compassion (63) in men populations can be used to interrupt guilt-rumination cycles, and emotional assertiveness training (64) in women can be used to address regret around emotional expression.

### 4.2 Limitations

Some limitations should be noted. First, because the current study is a cross-sectional study, it cannot establish causal relationships in the interpretation of findings despite the fact that the use of a network analytic approach based on partial correlation networks is statistically approximate. Future research should employ longitudinal or experimental designs, and temporal network models (e.g., vector autoregressive models) may particularly benefit from examining dynamic, time-lagged relationships between symptoms and more robustly assessing causal dynamics. Second, during the case-dropping bootstrap procedure, the instability of bridge EI centrality across all three networks limits its interpretability and warrants cautious interpretation. For further study, large-scale and multi-center studies may be able to address this shortcoming. Thirdly, although this study proposed targeting bridging symptoms through cognitive control training (54) or mindfulness-based interventions (56) as a potential means of linking dissociative depressive symptoms with ambivalence over emotional expression, the feasibility of achieving such dissociative effects remains uncertain. Given that no interventions were implemented, this warrants further interventions, including simulation-based network analysis (65). Fourth, the sample consisted of relatively homogeneous college students, which may limit the generalizability of the findings to clinical populations, older adults, or individuals from cultures with different norms for emotional expression. Replication with more diverse and cross-cultural samples is necessary to enhance external validity. Additionally, response biases such as measurement error or social desirability in self-report questionnaires may have influenced the reporting of symptoms. Such biases could be addressed in future research by employing multimethod assessment approaches, such as clinician ratings, behavioral tasks, or physiological indicators. Finally, gender was assessed using a binary measure (“man” or “woman”) based on participants’ self-identification. We did not collect data on individuals who identify outside this binary (e.g., non-binary, genderqueer, or other gender identities). This binary categorization may limit the inclusiveness and generalizability of the findings, particularly for populations with more diverse gender identities. Future research should incorporate more comprehensive gender assessments to better capture the experiences of individuals across the full gender spectrum.

## 5 Conclusion

The current study employed a network approach to examine the interrelation between depressive symptoms and ambivalence over emotional expression among college students. The results revealed that the edge between “*concentration difficulties*” and “*emotional rumination*”, as well as the edge between “*guilt*” and “*regret expressing*”, exhibited the strongest association that linked depression and ambivalence over emotional expression. Furthermore, “*guilt*” is the common bridging symptom in the men’s and women’s networks. Meanwhile, “*emotional rumination*” and “*regret expressing*” were identified as the main bridging symptoms within men’s and women’s networks, respectively. In summary, our study’s findings imply that cognitive control training or mindfulness-based interventions that focus on bridging symptoms may disassociate the interrelation between depression and ambivalence over emotional expression.

## Data Availability

The raw data supporting the conclusions of this article will be made available by the authors, without undue reservation.
