# Supplementary material for: The interrelation between major depressive symptoms and ambivalence over emotional expression among college students: a network perspective on gender differences
Source: Front Psychiatry. 2025 Sep 17;16:1658159. doi: 10.3389/fpsyt.2025.1658159 (PMC12484036; doi:10.3389/fpsyt.2025.1658159)
Supplement: Supplementary file 1 [file Supplementaryfile1.docx]

**Online Supplemental Materials**

**Tables**

**Table S1: The Edge-Weight Matrix and Standardized Centrality in the Depression-AEQ Network in the Total Sample.**

| **Node** |  | **Edge-weight matrix** | | | | | | | | | | | | | |  | **Standardized centrality** | | | |
| --- | --- | --- | --- | --- | --- | --- | --- | --- | --- | --- | --- | --- | --- | --- | --- | --- | --- | --- | --- | --- |
|  |  | PHQ1 | PHQ2 | PHQ3 | PHQ4 | PHQ5 | PHQ6 | PHQ7 | PHQ8 | PHQ9 | AEQ1 | AEQ2 | AEQ3 | AEQ4 | AEQ5 |  | EI | | Bridge EI | |
| **Depression** |  |  |  |  |  |  |  |  |  |  |  |  |  |  |  |  |  |  |  |  |
| PHQ1: Anhedonia |  | 0 | 0.304 | 0.060 | 0.194 | 0.097 | 0.084 | 0 | 0 | 0 | 0 | 0 | 0.039 | 0 | 0 |  | -0.552 |  | 0.403 |  |
| PHQ2: Depressed or sad mood |  | 0.304 | 0 | 0.140 | 0.088 | 0 | 0.146 | 0 | 0.123 | 0.142 | 0 | 0.047 | 0 | 0 | 0 |  | 0.724 |  | 0.703 |  |
| PHQ3: Sleep difficulties |  | 0.060 | 0.140 | 0 | 0.326 | 0.205 | 0 | 0.059 | 0 | 0 | 0 | 0 | 0 | 0 | 0 |  | -0.472 |  | -1.085 |  |
| PHQ4: Fatigue |  | 0.194 | 0.088 | 0.326 | 0 | 0.104 | 0 | 0.156 | 0 | 0 | 0 | 0 | 0 | 0 | 0 |  | -0.006 |  | -1.085 |  |
| PHQ5: Appetite changes |  | 0.097 | 0 | 0.205 | 0.104 | 0 | 0.096 | 0.134 | 0.119 | 0 | 0 | 0 | 0 | 0 | 0 |  | -0.689 |  | -1.085 |  |
| PHQ6: Guilt |  | 0.084 | 0.146 | 0 | 0 | 0.096 | 0 | 0.294 | 0.079 | 0.177 | 0 | 0 | 0 | 0 | 0.055 |  | 0.372 |  | 1.030 |  |
| PHQ7: Concentration difficulties |  | 0 | 0 | 0.059 | 0.156 | 0.134 | 0.294 | 0 | 0.294 | 0 | 0.058 | 0 | 0 | 0 | 0 |  | 0.751 |  | 1.119 |  |
| PHQ8: Motor disturbances |  | 0 | 0.123 | 0 | 0 | 0.119 | 0.079 | 0.294 | 0 | 0.207 | 0 | 0 | 0 | 0 | 0 |  | -0.282 |  | -1.085 |  |
| PHQ9: Suicide ideation |  | 0 | 0.142 | 0 | 0 | 0 | 0.177 | 0 | 0.207 | 0 | 0 | 0 | 0 | 0 | 0 |  | -2.062 |  | -1.085 |  |
| **AEQ** |  |  |  |  |  |  |  |  |  |  |  |  |  |  |  |  |  |  |  |  |
| AEQ1: Emotional rumination |  | 0 | 0 | 0 | 0 | 0 | 0 | 0.058 | 0 | 0 | 0 | 0.427 | 0.098 | 0 | 0.103 |  | -1.102 |  | 1.119 |  |
| AEQ2: Desire to be understood |  | 0 | 0.047 | 0 | 0 | 0 | 0 | 0 | 0 | 0 | 0.427 | 0 | 0.148 | 0.339 | 0.207 |  | 1.792 |  | 0.703 |  |
| AEQ3: Inhibit positive emotion expression |  | 0.039 | 0 | 0 | 0 | 0 | 0 | 0 | 0 | 0 | 0.098 | 0.148 | 0 | 0.500 | 0.244 |  | 0.955 |  | 0.403 |  |
| AEQ4: Inhibit negative emotion expression |  | 0 | 0 | 0 | 0 | 0 | 0 | 0 | 0 | 0 | 0 | 0.339 | 0.500 | 0 | 0.193 |  | 0.971 |  | -1.085 |  |
| AEQ5: Regret expressing |  | 0 | 0 | 0 | 0 | 0 | 0.055 | 0 | 0 | 0 | 0.103 | 0.207 | 0.244 | 0.193 | 0 |  | -0.399 |  | 1.030 |  |

***Note*.** EI, Expected Influence. Bridge EI, bridge Expected Influence.

**Table S2: The Edge-Weight Matrix and Standardized Centrality in the Depression-AEQ Network in the Male Sample.**

| **Node** |  | **Edge-weight matrix** | | | | | | | | | | | | | |  | **Standardized centrality** | | | |
| --- | --- | --- | --- | --- | --- | --- | --- | --- | --- | --- | --- | --- | --- | --- | --- | --- | --- | --- | --- | --- |
|  |  | PHQ1 | PHQ2 | PHQ3 | PHQ4 | PHQ5 | PHQ6 | PHQ7 | PHQ8 | PHQ9 | AEQ1 | AEQ2 | AEQ3 | AEQ4 | AEQ5 |  | EI | | Bridge EI | |
| **Depression** |  |  |  |  |  |  |  |  |  |  |  |  |  |  |  |  |  |  |  |  |
| PHQ1: Anhedonia |  | 0 | 0.363 | 0 | 0.176 | 0 | 0 | 0 | 0.114 | 0 | 0 | 0 | 0 | 0 | 0 |  | -0.976 |  | -0.393 |  |
| PHQ2: Depressed or sad mood |  | 0.363 | 0 | 0.153 | 0.109 | 0 | 0.112 | 0 | 0 | 0.149 | 0 | 0 | 0 | 0 | 0 |  | 0.375 |  | -0.393 |  |
| PHQ3: Sleep difficulties |  | 0 | 0.153 | 0 | 0.345 | 0.188 | 0 | 0 | 0 | 0 | 0 | 0 | 0 | 0 | 0 |  | -0.789 |  | -0.393 |  |
| PHQ4: Fatigue |  | 0.176 | 0.109 | 0.345 | 0 | 0.131 | 0 | 0.147 | 0 | 0 | 0 | 0 | 0 | 0 | 0 |  | 0.495 |  | -0.393 |  |
| PHQ5: Appetite changes |  | 0 | 0 | 0.188 | 0.131 | 0 | 0.140 | 0.105 | 0.094 | 0 | 0 | 0 | 0 | 0 | 0 |  | -0.957 |  | -0.393 |  |
| PHQ6: Guilt |  | 0 | 0.112 | 0 | 0 | 0.140 | 0 | 0.304 | 0.146 | 0.152 | 0.076 | 0 | 0 | 0 | 0 |  | 0.623 |  | 2.360 |  |
| PHQ7: Concentration difficulties |  | 0 | 0 | 0 | 0.147 | 0.105 | 0.304 | 0 | 0.268 | 0 | 0 | 0 | 0 | 0 | 0 |  | 0.012 |  | -0.393 |  |
| PHQ8: Motor disturbances |  | 0.114 | 0 | 0 | 0 | 0.094 | 0.146 | 0.268 | 0 | 0.217 | 0 | 0 | 0 | 0 | 0 |  | 0.095 |  | -0.393 |  |
| PHQ9: Suicide ideation |  | 0 | 0.149 | 0 | 0 | 0 | 0.152 | 0 | 0.217 | 0 | 0 | 0 | 0 | 0 | 0 |  | -1.757 |  | -0.393 |  |
| **AEQ** |  |  |  |  |  |  |  |  |  |  |  |  |  |  |  |  |  |  |  |  |
| AEQ1: Emotional rumination |  | 0 | 0 | 0 | 0 | 0 | 0.076 | 0 | 0 | 0 | 0 | 0.422 | 0.084 | 0 | 0.113 |  | -0.735 |  | 2.360 |  |
| AEQ2: Desire to be understood |  | 0 | 0 | 0 | 0 | 0 | 0 | 0 | 0 | 0 | 0.422 | 0 | 0.146 | 0.352 | 0.190 |  | 1.664 |  | -0.393 |  |
| AEQ3: Inhibit positive emotion expression |  | 0 | 0 | 0 | 0 | 0 | 0 | 0 | 0 | 0 | 0.084 | 0.146 | 0 | 0.530 | 0.289 |  | 1.311 |  | -0.393 |  |
| AEQ4: Inhibit negative emotion expression |  | 0 | 0 | 0 | 0 | 0 | 0 | 0 | 0 | 0 | 0 | 0.352 | 0.530 | 0 | 0.140 |  | 1.158 |  | -0.393 |  |
| AEQ5: Regret expressing |  | 0 | 0 | 0 | 0 | 0 | 0 | 0 | 0 | 0 | 0.113 | 0.190 | 0.289 | 0.140 | 0 |  | -0.519 |  | -0.393 |  |

***Note*.** EI, Expected Influence. Bridge EI, bridge Expected Influence.

**Table S3: The Edge-Weight Matrix and Standardized Centrality in the Depression-AEQ Network in the Female Sample.**

| **Node** |  | **Edge-weight matrix** | | | | | | | | | | | | | |  | **Standardized centrality** | | | |
| --- | --- | --- | --- | --- | --- | --- | --- | --- | --- | --- | --- | --- | --- | --- | --- | --- | --- | --- | --- | --- |
|  |  | PHQ1 | PHQ2 | PHQ3 | PHQ4 | PHQ5 | PHQ6 | PHQ7 | PHQ8 | PHQ9 | AEQ1 | AEQ2 | AEQ3 | AEQ4 | AEQ5 |  | EI | | Bridge EI | |
| **Depression** |  |  |  |  |  |  |  |  |  |  |  |  |  |  |  |  |  |  |  |  |
| PHQ1: Anhedonia |  | 0 | 0.247 | 0 | 0.213 | 0.135 | 0.108 | 0 | 0 | 0 | 0 | 0 | 0.060 | 0 | 0 |  | -0.446 |  | 0.790 |  |
| PHQ2: Depressed or sad mood |  | 0.247 | 0 | 0.128 | 0 | 0 | 0.177 | 0 | 0.205 | 0.143 | 0 | 0.060 | 0 | 0 | 0 |  | 0.627 |  | 0.790 |  |
| PHQ3: Sleep difficulties |  | 0 | 0.128 | 0 | 0.307 | 0.211 | 0 | 0 | 0 | 0 | 0 | 0 | 0 | 0 | 0 |  | -1.079 |  | -0.803 |  |
| PHQ4: Fatigue |  | 0.213 | 0 | 0.307 | 0 | 0 | 0 | 0.172 | 0.097 | 0 | 0 | 0 | 0 | 0 | 0 |  | -0.307 |  | -0.803 |  |
| PHQ5: Appetite changes |  | 0.135 | 0 | 0.211 | 0 | 0 | 0 | 0.163 | 0.137 | 0 | 0 | 0 | 0 | 0 | 0 |  | -1.084 |  | -0.803 |  |
| PHQ6: Guilt |  | 0.108 | 0.177 | 0 | 0 | 0 | 0 | 0.294 | 0 | 0.195 | 0 | 0 | 0 | 0 | 0.092 |  | 0.116 |  | 1.634 |  |
| PHQ7: Concentration difficulties |  | 0 | 0 | 0 | 0.172 | 0.163 | 0.294 | 0 | 0.322 | 0 | 0 | 0 | 0 | 0 | 0 |  | 0.576 |  | -0.803 |  |
| PHQ8: Motor disturbances |  | 0 | 0.205 | 0 | 0.097 | 0.137 | 0 | 0.322 | 0 | 0.196 | 0 | 0 | 0 | 0 | 0 |  | 0.605 |  | -0.803 |  |
| PHQ9: Suicide ideation |  | 0 | 0.143 | 0 | 0 | 0 | 0.195 | 0 | 0.196 | 0 | 0 | 0 | 0 | 0 | 0 |  | -1.688 |  | -0.803 |  |
| **AEQ** |  | 0 | 0 | 0 | 0 | 0 | 0 | 0 | 0 | 0 | 0 | 0.419 | 0.094 | 0.053 | 0.081 |  | -1.079 |  | -0.803 |  |
| AEQ1: Emotional rumination |  | 0 | 0.060 | 0 | 0 | 0 | 0 | 0 | 0 | 0 | 0.419 | 0 | 0.155 | 0.312 | 0.229 |  | 1.792 |  | 0.790 |  |
| AEQ2: Desire to be understood |  | 0.060 | 0 | 0 | 0 | 0 | 0 | 0 | 0 | 0 | 0.094 | 0.155 | 0 | 0.465 | 0.203 |  | 0.721 |  | 0.790 |  |
| AEQ3: Inhibit positive emotion expression |  | 0 | 0 | 0 | 0 | 0 | 0 | 0 | 0 | 0 | 0.053 | 0.312 | 0.465 | 0 | 0.243 |  | 1.232 |  | -0.803 |  |
| AEQ4: Inhibit negative emotion expression |  | 0 | 0 | 0 | 0 | 0 | 0.092 | 0 | 0 | 0 | 0.081 | 0.229 | 0.203 | 0.243 | 0 |  | 0.017 |  | 1.634 |  |
| AEQ5: Regret expressing |  | 0 | 0.247 | 0 | 0.213 | 0.135 | 0.108 | 0 | 0 | 0 | 0 | 0 | 0.060 | 0 | 0 |  | -0.446 |  | 0.790 |  |

***Note*.** EI, Expected Influence. Bridge EI, bridge Expected Influence.

**Table S4: Gender Differences in the Edges in Depression-AEQ Networks.**

| **ID** | **Undirected edge** | **Edge weight** | | ***Diff*** | ***p*** | |
| --- | --- | --- | --- | --- | --- | --- |
|  |  | **Male** | **Female** |  |  |  |
| 1 | AEQ1 - AEQ2 | 0.422 | 0.419 | 0.004 | .931 |  |
| 2 | AEQ1 - AEQ3 | 0.084 | 0.094 | 0.010 | .881 |  |
| 3 | AEQ1 - AEQ4 | 0.000 | **0.053** | 0.053 | .010 | ** |
| 4 | AEQ1 - AEQ5 | 0.113 | 0.081 | 0.032 | .554 |  |
| 5 | AEQ2 - AEQ3 | 0.146 | 0.155 | 0.010 | .812 |  |
| 6 | AEQ2 - AEQ4 | 0.352 | 0.312 | 0.040 | .485 |  |
| 7 | AEQ2 - AEQ5 | 0.190 | 0.229 | 0.039 | .594 |  |
| 8 | AEQ3 - AEQ4 | 0.530 | 0.465 | 0.066 | .277 |  |
| 9 | AEQ3 - AEQ5 | 0.289 | 0.203 | 0.086 | .109 |  |
| 10 | AEQ4 - AEQ5 | 0.140 | 0.243 | 0.102 | .188 |  |
| 11 | PHQ1 - PHQ2 | 0.363 | 0.247 | 0.116 | .129 |  |
| 12 | PHQ1 - PHQ4 | 0.176 | 0.213 | 0.037 | .485 |  |
| 13 | PHQ1 - PHQ5 | 0.000 | 0.135 | 0.135 | .267 |  |
| 14 | PHQ1 - PHQ6 | 0.000 | 0.108 | 0.108 | .515 |  |
| 15 | PHQ1 - PHQ8 | **0.114** | 0.000 | 0.114 | .079 | † |
| 16 | PHQ1 - AEQ3 | 0.000 | 0.060 | 0.060 | .356 |  |
| 17 | PHQ2 - PHQ3 | 0.153 | 0.128 | 0.025 | .733 |  |
| 18 | PHQ2 - PHQ4 | 0.109 | 0.000 | 0.109 | .584 |  |
| 19 | PHQ2 - PHQ6 | 0.112 | 0.177 | 0.065 | .287 |  |
| 20 | PHQ2 - PHQ8 | 0.000 | **0.205** | 0.205 | .059 | † |
| 21 | PHQ2 - PHQ9 | 0.149 | 0.143 | 0.006 | .950 |  |
| 22 | PHQ2 - AEQ2 | 0.000 | 0.060 | 0.060 | .426 |  |
| 23 | PHQ3 - PHQ4 | 0.345 | 0.307 | 0.038 | .564 |  |
| 24 | PHQ3 - PHQ5 | 0.188 | 0.211 | 0.024 | .723 |  |
| 25 | PHQ4 - PHQ5 | 0.131 | 0.000 | 0.131 | .535 |  |
| 26 | PHQ4 - PHQ7 | 0.147 | 0.172 | 0.025 | .653 |  |
| 27 | PHQ4 - PHQ8 | 0.000 | 0.097 | 0.097 | .139 |  |
| 28 | PHQ5 - PHQ6 | 0.140 | 0.000 | 0.140 | .178 |  |
| 29 | PHQ5 - PHQ7 | 0.105 | 0.163 | 0.058 | .257 |  |
| 30 | PHQ5 - PHQ8 | 0.094 | 0.137 | 0.043 | .624 |  |
| 31 | PHQ6 - PHQ7 | 0.304 | 0.294 | 0.010 | .901 |  |
| 32 | PHQ6 - PHQ8 | 0.146 | 0.000 | 0.146 | .119 |  |
| 33 | PHQ6 - PHQ9 | 0.152 | 0.195 | 0.043 | .663 |  |
| 34 | PHQ6 - AEQ1 | 0.076 | 0.000 | 0.076 | .198 |  |
| 35 | PHQ6 - AEQ5 | 0.000 | **0.092** | 0.092 | .030 | * |
| 36 | PHQ7 - PHQ8 | 0.268 | 0.322 | 0.054 | .416 |  |
| 37 | PHQ8 - PHQ9 | 0.217 | 0.196 | 0.021 | .743 |  |

***Note.*** Stronger edges were marked in highlighted in bold. † *p* < .1, * *p* < .05, ** *p* < .01.

**Table S5: Gender Differences in the Centrality of Nodes in Depression-AEQ Networks.**

| **Node** | **EI** | | | | | |  | **Bridge EI** | | | | | |
| --- | --- | --- | --- | --- | --- | --- | --- | --- | --- | --- | --- | --- | --- |
|  | **Male** | **Female** | **diff** | | ***p*** | |  | **Male** | **Female** | **diff** | | ***p*** | |
| **Depression** |  |  |  |  |  |  |  |  |  |  |  |  |  |
| PHQ1: Anhedonia | 0.654 | 0.763 | -0.110 |  | .545 |  |  | 0.000 | 0.060 | -0.060 |  | .347 |  |
| PHQ2: Depressed or sad mood | 0.888 | 0.961 | -0.073 |  | .663 |  |  | 0.000 | 0.060 | -0.060 |  | .406 |  |
| PHQ3: Sleep difficulties | 0.686 | 0.647 | 0.040 |  | .703 |  |  | 0.000 | 0.000 | 0.000 |  | 1.000 |  |
| PHQ4: Fatigue | 0.908 | 0.789 | 0.120 |  | .406 |  |  | 0.000 | 0.000 | 0.000 |  | 1.000 |  |
| PHQ5: Appetite changes | 0.657 | 0.646 | 0.011 |  | .960 |  |  | 0.000 | 0.000 | 0.000 |  | 1.000 |  |
| PHQ6: Guilt | 0.930 | 0.867 | 0.064 |  | .802 |  |  | 0.076 | 0.092 | -0.016 |  | .653 |  |
| PHQ7: Concentration difficulties | 0.825 | 0.952 | -0.127 |  | .218 |  |  | 0.000 | 0.000 | 0.000 |  | 1.000 |  |
| PHQ8: Motor disturbances | 0.839 | 0.957 | -0.118 |  | .485 |  |  | 0.000 | 0.000 | 0.000 |  | 1.000 |  |
| PHQ9: Suicide ideation | 0.518 | 0.534 | -0.016 |  | .891 |  |  | 0.000 | 0.000 | 0.000 |  | 1.000 |  |
| **AEQ** |  |  |  |  |  |  |  |  |  |  |  |  |  |
| AEQ1: Emotional rumination | 0.695 | 0.646 | 0.049 |  | .535 |  |  | 0.076 | 0.000 | 0.076 |  | .257 |  |
| AEQ2: Desire to be understood | 1.111 | 1.176 | -0.065 |  | .475 |  |  | 0.000 | 0.060 | -0.060 |  | .406 |  |
| AEQ3: Inhibit positive emotion expression | 1.050 | 0.978 | 0.071 |  | .376 |  |  | 0.000 | 0.060 | -0.060 |  | .337 |  |
| AEQ4: Inhibit negative emotion expression | 1.023 | 1.072 | -0.049 |  | .495 |  |  | 0.000 | 0.000 | 0.000 |  | 1.000 |  |
| AEQ5: Regret expressing | 0.733 | 0.848 | -0.116 |  | .218 |  |  | 0.000 | **0.092** | -0.092 |  | **.040** | * |

***Note.*** Higher centrality was marked in highlighted in bold. * *p* < .05.

**Figures**





**Figure S1: Bootstrapped Accuracy of Edge Weights in the Depression-AEQ Network for the Total Sample (A), Male Sample (B), and Female Sample (C).**

***Note*.** the red line indicates the estimated edge, and the dark area indicates the 95% bootstrap CI.


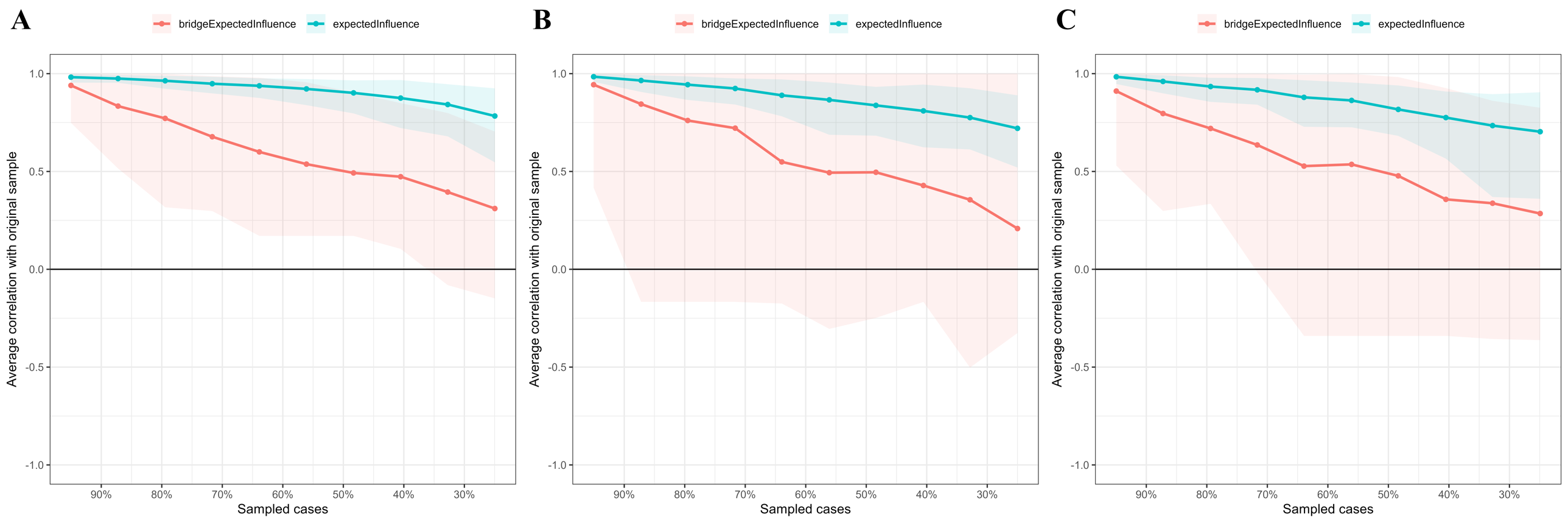


**Figure S2: Bootstrapped Stability of Centrality Estimates in the Depression-AEQ Network for the Total Sample (A), Male Sample (B), and Female Sample (C).**

***Note*.** **y-axis**: Mean correlation between centrality metrics from the original network and those from networks re-estimated after progressively removing larger proportions of the sample. Correlation exceeding 0.25, 0.5, and 0.75 reflects acceptable, good, and excellent stability, respectively.





**Figure S3: Bootstrapped Differences in Edge Weights in the Depression-AEQ Network for the Total Sample (A), Male Sample (B), and Female Sample (C).**

***Note*.** Box colors indicate whether edge weights differ significantly: black denotes significant differences, while gray indicates no significant difference. The diagonal line reflects edge strength, with colors ranging from red (negative associations) to white (weaker edges) to blue (stronger positive connections).





**Figure S4: Bootstrapped Differences in Centrality Values Between Nodes in the Depression-AEQ Network for the Total Sample (A & B), Male Sample (C & D), and Female Sample (E & F).**

***Note*.** Box colors indicate whether edge weights differ significantly: black denotes significant differences, while gray indicates no significant difference. The numbers in the white boxes (i.e., diagonal line) represent the values of the nodes’ raw centrality.
